# Supplementary material for: Semantic Harmonization of Alzheimer’s Disease Datasets Using AD-Mapper
Source: J Alzheimers Dis. 2024 Jun 11;99(4):1409–23. doi: 10.3233/JAD-240116 (PMC11191441; doi:10.3233/JAD-240116)
Supplement: Supplementary Material [file jad-99-jad240116-s001.pdf]

Supplementary Material

Semantic Harmonization of Alzheimer’s Disease Datasets Using AD-Mapper

Supplementary Table 1. The total number of collected variables in BRACE, AMED, and ALFA studies as well as the number of variables (i.e., the number of variables that could be harmonized against the AD-Mapper).

Dataset	Consortium	# Overlapping variables	# Total variables
BRACE [1]	Bristol Research into Alzheimer's and Care for the Elderly	34	476
AMED [2]	The Japanese Agency for Medical Research and Development	44	691
ALFA [3]	For Alzheimer and Families	53	252

Supplementary Table 2. Total number of mapped variables in the extended AD-Mapper CDM.

**Variable origin**		**# Mapped variables**
**Cohort**	**A4** [4]	97
	**ABVIB** [5]	12
	**ADNI** [6]	413
	**AIBL** [7]	58
	**ALFA** [3]	56
	**AMED** [2]	105
	**ANM** [8]	162
	**ARWIBO** [9]	1129
	**BRACE** [1]	36
	**DOD-ADNI** [10]	395
	**EDSD** [11]	1061
	**EMIF** [12]	31
	**EPAD** [13]	140
	**I-ADNI** [14]	1070
	**JADNI** [15]	720
	**NACC** [16]	229
	**OASIS** [17]	1057
	**PREVENT-AD** [18]	35
	**PharmaCog** [19]	1073
	**ROSMAP** [20]	30
	**VASCULAR** [21]	55
	**VITA** [22]	1054
	**WMH-AD** [23]	1054
**CDM**	**NEURO Cohort**	14
	**C-Surv** [24]	47
	**OMOP** [25]	144
**Other**	**CURIE** [26]	218
	**Reference term**	1300

Supplementary Table 3. Performance scores of utilizing the Model B alone. K indicates the number of candidates to be assessed by the model.

**Dataset**	**K**	**Accuracy (in %)**
CDM test set	5	82.93
	10	84.83
BRACE	5	76.47
	10	76.47
AMED	5	79.54
	10	79.54
ALFA	5	71.69
	10	71.69

Variables that fall outside the AD-Mapper common data model (CDM)

Since cohort studies were conducted to address specific research questions, the measurements collected often vary from one cohort to another. In the AD-Mapper CDM, our goal was to include variables that were common in at least two cohorts. Similarly, as mentioned in the main manuscript, the total number of included variables in the external CDMs was limited. Consequently, the variable naming space of our CDM was limited to those cohorts and CDMs. Given this challenge, a highly relevant question was how the model would handle variables that were not initially part of the AD-Mapper CDM and had not yet been incorporated into the learned embedding space. To assess the pipeline's performance in this scenario, we manually mapped 82 variables from the ADNI cohort and added them to the AD-Mapper CDM, after which they were integrated into the embedding space. Finally, we conducted an experiment to evaluate the model's accuracy in mapping variables that were added to the model at a later stage. For this analysis, we used Model A (classifier) to estimate the accuracy of harmonizing the Alzheimer’s Disease Neuroimaging Initiative (ADNI) cohort. Subsequently, we performed a similar analysis using the string-matching technique (Supplementary Table 3). Our results revealed an accuracy of 48.1% compared to the string-matching accuracy of 12.1%.

Supplementary Table 4. Performance on completely unseen variables of ADNI harmonized using the AD-mapper and string-matching techniques.

**Model**	**W-values (W_1, W_2)**	**K**	**Accuracy (in %)**
String-matching	-	-	12.1
AD-Mapper	1.0,0.0	82	48.05

Importance of data dictionary descriptiveness

As described in the main manuscript, we utilized the mappings of different cohorts and CDMs to generate a training dataset. We included variable descriptions wherever available to enhance the model's comprehension of the variables' semantics. This step was taken because variables in cohort studies frequently lacked descriptiveness, and by adding these descriptions, the model could potentially identify similarities between the input variables and the reference terms. Similarly, when the model was used for harmonization of the new cohort studies (i.e., previously unseen by the model and thus, excluded from the AD-Mapper CDM), the variable descriptions played a significant role. However, in the version of the AMED cohort's data dictionary that we retrieved, the variable descriptions were not readable and displayed question marks, possibly due to a formatting issue. Considering the similarities between the variable naming convention of AMED and those of ADNI, JADNI, I-ADNI, and DOD-ADNI cohorts, the model could potentially have performed better if the descriptions for all variables in the AMED cohort were available.

REFERENCES

[1] Tales A (2019) BRACE [Data set]. Dementias Platform UK. https://doi.org/10.48532/008000

[2] Suzuki K (2017) Preclinical AD and Biomarker; from J-ADNI to AMED Preclinical Study. *Brain Nerve* **69**, 691–700.

[3] Molinuevo JL, Gramunt N, Gispert JD, Fauria K, Esteller M, Minguillon C, Sánchez-Benavides G, Huesa G, Morán S, Dal-Ré R, Camí J (2016) The ALFA project: a research platform to identify early pathophysiological features of Alzheimer's disease. *Alzheimers Dement (N Y)* **2**, 82-92.

[4] Sperling RA, Rentz DM, Johnson KA, Karlawish J, Donohue M, Salmon DP, Aisen P (2014) The A4 study: stopping AD before symptoms begin?. *Sci Transl Med* **6**, 228fs13.

[5] Rodriguez, FS, Zheng, L, Chui, HC, Aging Brain: Vasculature, Ischemia, and Behavior Study (2019) Psychometric characteristics of cognitive reserve: how high education might improve certain cognitive abilities in aging. *Dement Geriatr Cogn Disord* **47**, 335-344.

[6] Mueller SG, Weiner MW, Thal LJ, Petersen RC, Jack CR, Jagust W, Trojanowski JQ, Toga AW, Beckett L (2005) Ways toward an early diagnosis in Alzheimer’s disease: the Alzheimer’s Disease Neuroimaging Initiative (ADNI). *Alzheimers Dement* **1**, 55-66.

[7] Ellis KA, Bush AI, Darby D, De Fazio D, Foster J, Hudson P, Lautenschlager NT, Lenzo N, Martins RN, Maruff P, Masters C, Milner A, Pike K, Rowe C, Savage G, Szoeke C, Taddei K, Villemagne V, Woodward M, Ames D (2009) The Australian Imaging, Biomarkers and Lifestyle (AIBL) study of aging: methodology and baseline characteristics of 1112 individuals recruited for a longitudinal study of Alzheimer's disease. *Int Psychogeriatr* **21**, 672-687.

[8] Birkenbihl C, Westwood S, Shi L, Nevado-Holgado A, Westman E, Lovestone S, on behalf of the AddNeuroMed Consortium, Hofmann-Apitius M (2021) ANMerge: a comprehensive and accessible Alzheimer’s disease patient-level dataset. *J Alzheimers Dis* **79**, 423-431.

[9] Frisoni GB, Prestia A, Zanetti O, Galluzzi S, Romano M, Cotelli M, Gennarelli M, Binetti G, Bacchio L, Paghera B, Amicucci G, Bonetti M, Benussi L, Ghidoni R, Geroldi

C (2009) Markers of Alzheimer's disease in a population attending a memory clinic. *Alzheimers Dement* **5**, 307-317.

[10] Weiner MW, Veitch DP, Hayes J, Neylan T, Grafman J, Aisen PS, Petersen RC, Jack C, Jagust W, Trojanowski JQ, Shaw LM, Saykin AJ, Green RC, Harvey D, Toga AW, Friedl KE, Pacifico A, Sheline Y, Yaffe K, Mohlenoff B, Department of Defense Alzheimer's Disease Neuroimaging Initiative (2014) Effects of traumatic brain injury and posttraumatic stress disorder on Alzheimer's disease in veterans, using the Alzheimer's Disease Neuroimaging Initiative. *Alzheimers Dement* **10**, S226-S235.

[11] Brueggen K, Grothe MJ, Dyrba M, Fellgiebel A, Fischer F, Filippi M, Agosta F, Nestor P, Meisenzahl E, Blautzik J, Frölich L, Hausner L, Bokde ALW, Frisoni G, Pievani M, Klöppel S, Prvulovic D, Barkhof F, Pouwels PJW, Schröder J, Teipel S (2017) The European DTI Study on Dementia—a multicenter DTI and MRI study on Alzheimer's disease and mild cognitive impairment. *Neuroimage* **144**, 305-308.

[12] Bos I, Vos S, Vandenberghe R, Scheltens P, Engelborghs S, Frisoni G, Molinuevo JL, Wallin A, Lleó A, Popp J, Martinez-Lage P, Baird A, Dobson R, Legido-Quigley C, Sleegers K, Van Broeckhoven C, Bertram L, ten Kate M, Barkhof F, Zetterberg H, Lovestone S, Streffer J, Visser PJ (2018) The EMIF-AD Multimodal Biomarker Discovery study: design, methods and cohort characteristics. *Alzheimers Res Ther* **10**, 64.

[13] Solomon A, Kivipelto M, Molinuevo JL, Tom B, Ritchie CW (2018) European prevention of Alzheimer's dementia longitudinal cohort study (EPAD LCS): study protocol. *BMJ Open*, **8**, e021017.

[14] Cavedo E, Redolfi A, Angeloni F, Babiloni C, Lizio R, Chiapparini L, Bruzzone MG, Aquino D, Sabatini U, Alesiani M, Cherubini A, Salvatore E, Soricelli A, Vernieri F, Scrascia F, Sinforiani E, Chiarati P, Bastianello S, Montella P, Corbo D, Tedeschi G, Marino S, Baglieri A, De Salvo S, Carducci F, Quattrocchi CC, Cobelli M, Frisoni GB, for the Alzheimer's Disease Neuroimaging Initiative (2014) The Italian Alzheimer's Disease Neuroimaging Initiative (I-ADNI): Validation of Structural MR Imaging. *J Alzheimers Dis* **40**, 941-952.

[15] Iwatsubo T (2010) Japanese Alzheimer's Disease Neuroimaging Initiative: present status and future. *Alzheimers Dement* **6**, 297-299.

[16] Besser L, Kukull W, Knopman DS, Chui H, Galasko D, Weintraub S, Jicha G, Carlsson C, Bruns J, Quinn J, Sweet RA, Rascovsky K, Teylan M, Beekly D, Thomas G, Bollenbeck M, Monsell S, Mock C, Zhou XH, Thomas N, Robichaud E, Dean M, Hubbard J, Jacka M, Schwabe-Fry K, Wu J, the Neuropsychology Work Group, Directors, and Clinical Core leaders of the National Institute on Aging-funded US Alzheimer's Disease Centers, Phelps C, Morris JC (2018) Version 3 of the National Alzheimer's Coordinating Center's Uniform Data Set. *Alzheimer Dis Assoc Disord* **32**, 351.

[17] Marcus DS, Fotenos AF, Csernansky JG, Morris JC, Buckner RL (2010) Open access series of imaging studies: longitudinal MRI data in nondemented and demented older adults. *J Cogn Neurosci* **22**, 2677-2684.

[18] Breitner JCS, Poirier J, Etienne PE, Leoutsakos JM (2016) Rationale and Structure for a New Center for Studies on Prevention of Alzheimer's Disease (StoP-AD). *J Prev Alzheimers Dis* **3**, 236-242.

[19] Galluzzi S, Marizzoni M, Babiloni C, Albani D, Antelmi L, Bagnoli C, Bartes-Faz D, Cordone S, Didic M, Farotti L, Fiedler U, Forloni G, Girtler N, Hensch T, Jovicich J, Leeuwis A, Marra C, Molinuevo JL, Nobili F, Pariente J, Parnetti L, Payoux P, Del Percio C, Ranjeva J, Rolandi E, Rossini PM, Schönknecht P, Soricelli A, Tsolaki M, Visser PJ, Wiltfang J, Richardson JC, Bordet R, Blin O, Frisoni GB, the PharmaCog Consortium (2016) Clinical and biomarker profiling of prodromal Alzheimer's disease in workpackage 5 of the Innovative Medicines Initiative PharmaCog project: a 'European ADNI study'. *J Intern Med* **279**, 576-591.

[20] Bennett DA, Schneider JA, Arvanitakis Z, Wilson RS (2012) Overview and findings from the religious orders study. *Curr Alzheimer Res* **9**, 628-645.

[21] Emory University School of Medicine (2021, July). VASCULAR (VAScular ContribUtors to prodromaL AlzheimeR's disease). https://med.emory.edu/departments/medicine/divisions/geriatrics-gerontology/research/labs/bsharp/studies.html

[22] Fischer P, Jungwirth S, Krampla W, Weissgram S, Kirchmeyr W, Schreiber W, Huber K, Rainer M, Bauer P, Tragl KH (2002) *Vienna Transdanube Aging "VITA": study design, recruitment strategies and level of participation*. Springer, Vienna, pp. 105-116.

[23] Damulina A, Pirpamer L, Seiler S, Benke T, Dal-Bianco P, Ransmayr G, Struhal W, Hofer E, Langkammer C, Duering M, Fazekas F, Schmidt R (2019) White matter hyperintensities in Alzheimer's disease: a lesion probability mapping study. *J Alzheimers Dis* **68**, 789-796.

[24] Bauermeister S, Phatak M, Sparks K, Sargent L, Griswold M, McHugh C, Nalls M, Young S, Bauermeister J, Elliot P, Steptoe A, Porteous D, Dufouil C, Gallacher J (2023) Evaluating the harmonisation potential of diverse cohort datasets. *Eur J Epidemiol* **38**, 605-615.

[25] Observational Medical Outcomes Partnership. OMOP Common Data Model v5.4. Available from: https://athena.ohdsi.org/

[26] EBISPOT. OLS4 [webpage]. Hinxton: EMBL-EBI; [last updated 2023 Oct 10; cited 2023 Oct 10]. Available from: https://www.ebi.ac.uk/ols4.
